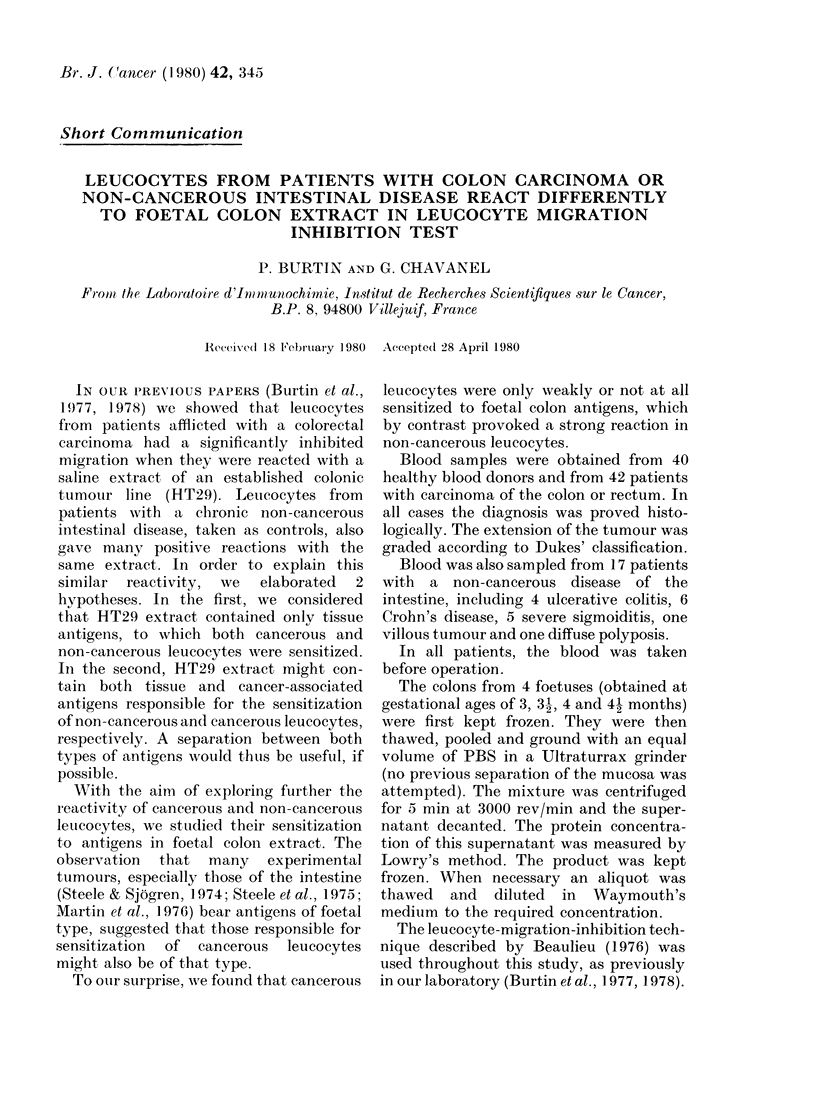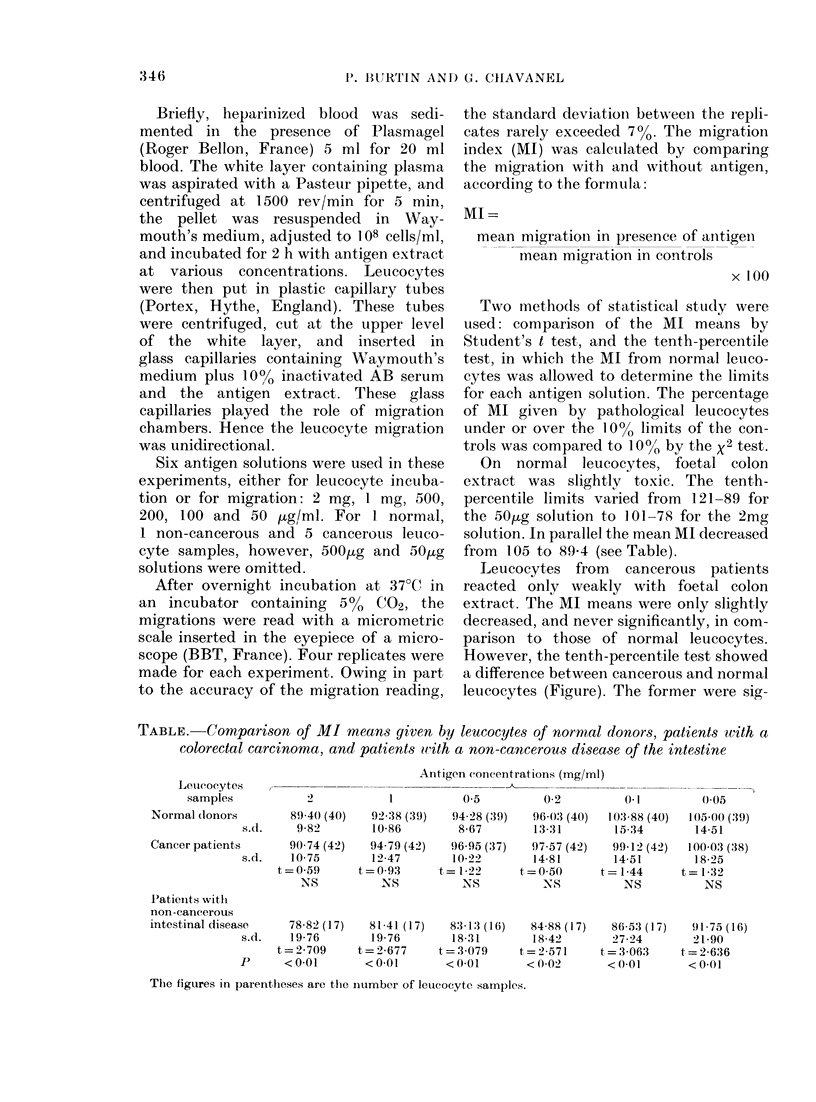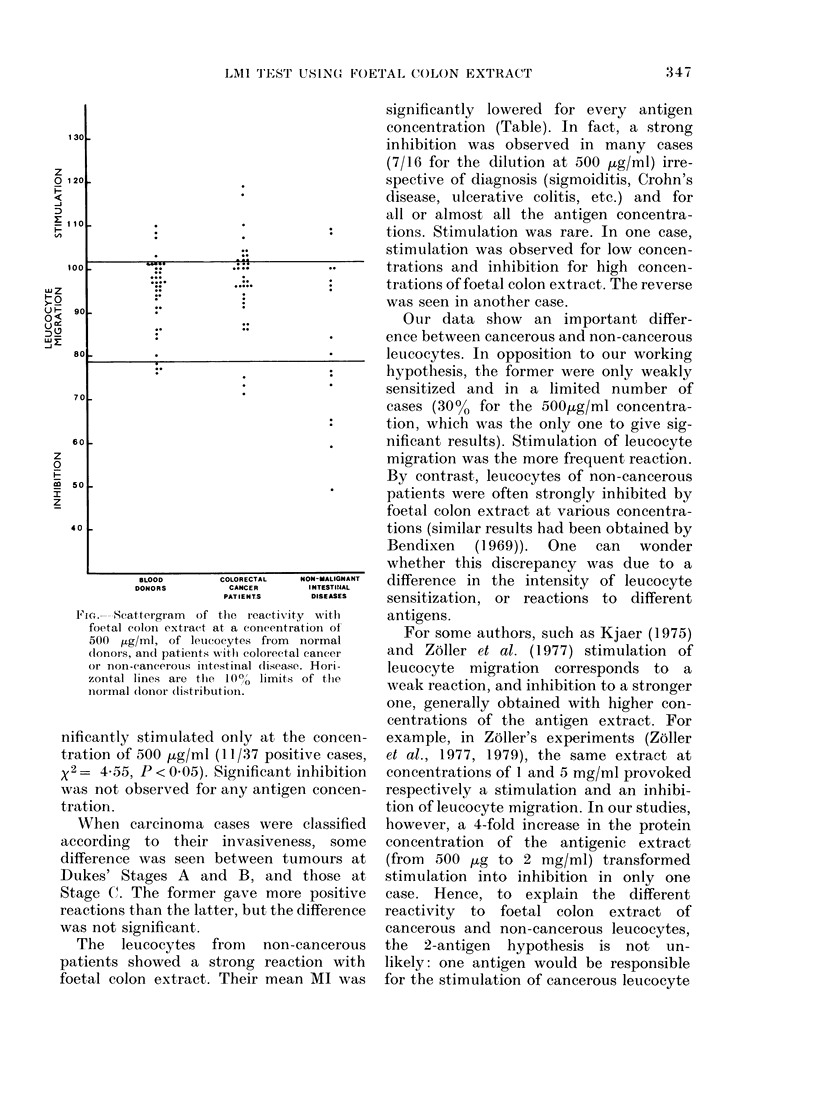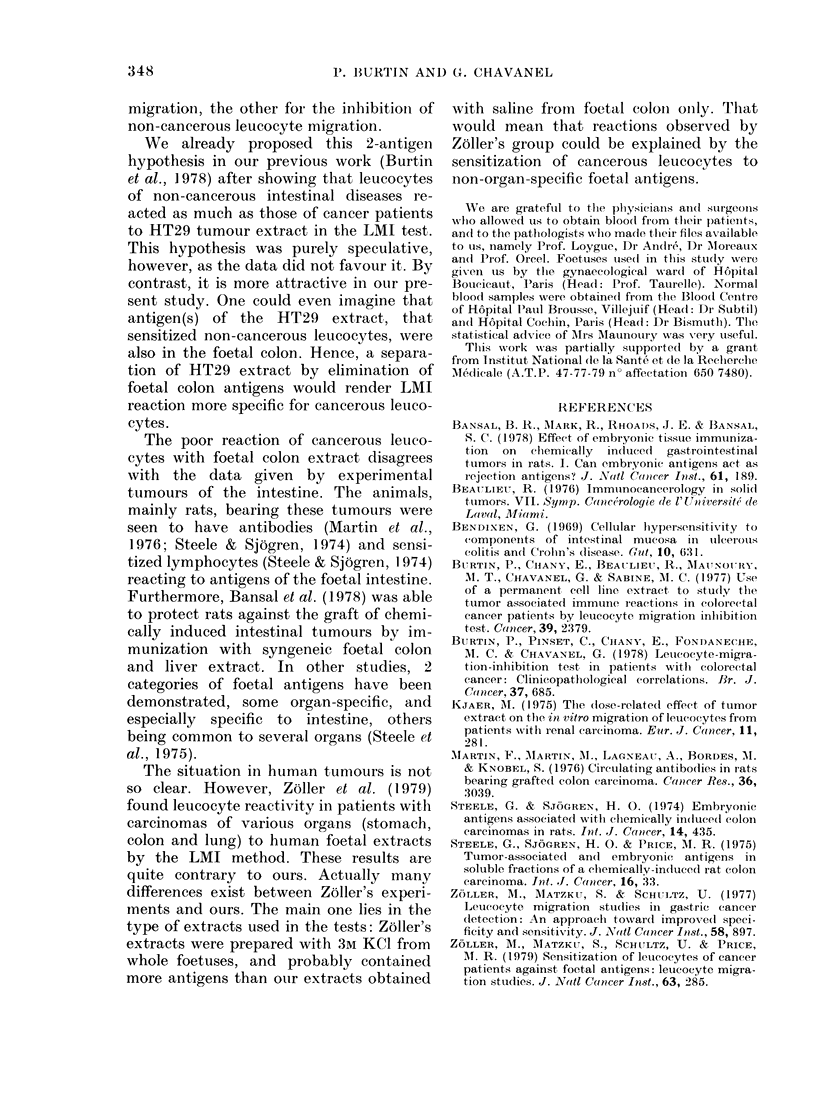# Leucocytes from patients with colon carcinoma or non-cancerous intestinal disease react differently to foetal colon extract in leucocyte migration inhibition test.

**DOI:** 10.1038/bjc.1980.237

**Published:** 1980-08

**Authors:** P. Burtin, G. Chavanel


*Br. J. Cancer* (1980) **42**, 345

***Short Communication***

### LEUCOCYTES FROM PATIENTS WITH COLON CARCINOMA OR NON-CANCEROUS INTESTINAL DISEASE REACT DIFFERENTLY TO FOETAL COLON EXTRACT IN LEUCOCYTE MIGRATION INHIBITION TEST

P. BURTIN AND G. CHAVANEL

*From the Laboratoire d'Immunochimie, Institut de Recherches Scientifiques sur le Cancer, B.P.* 8, 94800 *Villejuif, France*

Received 18 February 1980 Accepted 28 April 1980

IN OUR PREVIOUS PAPERS (Burtin *et al.*, 1977, 1978) we showed that leucocytes from patients afflicted with a colorectal carcinoma had a significantly inhibited migration when they were reacted with a saline extract of an established colonic tumour line (HT29). Leucocytes from patients with a chronic non-cancerous intestinal disease, taken as controls, also gave many positive reactions with the same extract. In order to explain this similar reactivity, we elaborated 2 hypotheses. In the first, we considered that HT29 extract contained only tissue antigens, to which both cancerous and non-cancerous leucocytes were sensitized. In the second, HT29 extract might contain both tissue and cancer-associated antigens responsible for the sensitization of non-cancerous and cancerous leucocytes, respectively. A separation between both types of antigens would thus be useful, if possible.

With the aim of exploring further the reactivity of cancerous and non-cancerous leucocytes, we studied their sensitization to antigens in foetal colon extract. The observation that many experimental tumours, especially those of the intestine (Steele & Sjögren, 1974; Steele *et al.*, 1975; Martin *et al.*, 1976) bear antigens of foetal type, suggested that those responsible for sensitization of cancerous leucocytes might also be of that type.

To our surprise, we found that cancerous leucocytes were only weakly or not at all sensitized to foetal colon antigens, which by contrast provoked a strong reaction in non-cancerous leucocytes.

Blood samples were obtained from 40 healthy blood donors and from 42 patients with carcinoma of the colon or rectum. In all cases the diagnosis was proved histologically. The extension of the tumour was graded according to Dukes' classification.

Blood was also sampled from 17 patients with a non-cancerous disease of the intestine, including 4 ulcerative colitis, 6 Crohn's disease, 5 severe sigmoiditis, one villous tumour and one diffuse polyposis.

In all patients, the blood was taken before operation.

The colons from 4 foetuses (obtained at gestational ages of 3, $3\frac{1}{2}$, 4 and $4\frac{1}{2}$ months) were first kept frozen. They were then thawed, pooled and ground with an equal volume of PBS in a Ultraturrax grinder (no previous separation of the mucosa was attempted). The mixture was centrifuged for 5 min at 3000 rev/min and the supernatant decanted. The protein concentration of this supernatant was measured by Lowry's method. The product was kept frozen. When necessary an aliquot was thawed and diluted in Waymouth's medium to the required concentration.

The leucocyte-migration-inhibition technique described by Beaulieu (1976) was used throughout this study, as previously in our laboratory (Burtin *et al.*, 1977, 1978).

Briefly, heparinized blood was sedimented in the presence of Plasmagel (Roger Bellon, France) 5 ml for 20 ml blood. The white layer containing plasma was aspirated with a Pasteur pipette, and centrifuged at 1500 rev/min for 5 min, the pellet was resuspended in Waymouth's medium, adjusted to $10^8$ cells/ml, and incubated for 2 h with antigen extract at various concentrations. Leucocytes were then put in plastic capillary tubes (Portex, Hythe, England). These tubes were centrifuged, cut at the upper level of the white layer, and inserted in glass capillaries containing Waymouth's medium plus 10% inactivated AB serum and the antigen extract. These glass capillaries played the role of migration chambers. Hence the leucocyte migration was unidirectional.

Six antigen solutions were used in these experiments, either for leucocyte incubation or for migration: 2 mg, 1 mg, 500, 200, 100 and 50 $\mu$g/ml. For 1 normal, 1 non-cancerous and 5 cancerous leucocyte samples, however, 500$\mu$g and 50$\mu$g solutions were omitted.

After overnight incubation at 37°C in an incubator containing 5% $CO_2$, the migrations were read with a micrometric scale inserted in the eyepiece of a microscope (BBT, France). Four replicates were made for each experiment. Owing in part to the accuracy of the migration reading, the standard deviation between the replicates rarely exceeded 7%. The migration index (MI) was calculated by comparing the migration with and without antigen, according to the formula:

$$MI = \frac{\text{mean migration in presence of antigen}}{\text{mean migration in controls}} \times 100$$

Two methods of statistical study were used: comparison of the MI means by Student's *t* test, and the tenth-percentile test, in which the MI from normal leucocytes was allowed to determine the limits for each antigen solution. The percentage of MI given by pathological leucocytes under or over the 10% limits of the controls was compared to 10% by the $\chi^2$ test.

On normal leucocytes, foetal colon extract was slightly toxic. The tenth-percentile limits varied from 121–89 for the 50$\mu$g solution to 101–78 for the 2mg solution. In parallel the mean MI decreased from 105 to 89·4 (see Table).

Leucocytes from cancerous patients reacted only weakly with foetal colon extract. The MI means were only slightly decreased, and never significantly, in comparison to those of normal leucocytes. However, the tenth-percentile test showed a difference between cancerous and normal leucocytes (Figure). The former were sig-

TABLE.—*Comparison of MI means given by leucocytes of normal donors, patients with a colorectal carcinoma, and patients with a non-cancerous disease of the intestine*

| Leucocytes samples | Antigen concentrations (mg/ml) 2 | 1 | 0·5 | 0·2 | 0·1 | 0·05 |
|---|---|---|---|---|---|---|
| Normal donors | 89·40 (40) | 92·38 (39) | 94·28 (39) | 96·03 (40) | 103·88 (40) | 105·00 (39) |
| s.d. | 9·82 | 10·86 | 8·67 | 13·31 | 15·34 | 14·51 |
| Cancer patients | 90·74 (42) | 94·79 (42) | 96·95 (37) | 97·57 (42) | 99·12 (42) | 100·03 (38) |
| s.d. | 10·75 | 12·47 | 10·22 | 14·81 | 14·51 | 18·25 |
| | t = 0·59 | t = 0·93 | t = 1·22 | t = 0·50 | t = 1·44 | t = 1·32 |
| | NS | NS | NS | NS | NS | NS |
| Patients with non-cancerous intestinal disease | 78·82 (17) | 81·41 (17) | 83·13 (16) | 84·88 (17) | 86·53 (17) | 91·75 (16) |
| s.d. | 19·76 | 19·76 | 18·31 | 18·42 | 27·24 | 21·90 |
| | t = 2·709 | t = 2·677 | t = 3·079 | t = 2·571 | t = 3·063 | t = 2·636 |
| *P* | < 0·01 | < 0·01 | < 0·01 | < 0·02 | < 0·01 | < 0·01 |

The figures in parentheses are the number of leucocyte samples.

FIG.—Scattergram of the reactivity with foetal colon extract at a concentration of 500 μg/ml, of leucocytes from normal donors, and patients with colorectal cancer or non-cancerous intestinal disease. Horizontal lines are the 10% limits of the normal donor distribution.

nificantly stimulated only at the concentration of 500 μg/ml (11/37 positive cases, $\chi^2 = 4{\cdot}55$, $P < 0{\cdot}05$). Significant inhibition was not observed for any antigen concentration.

When carcinoma cases were classified according to their invasiveness, some difference was seen between tumours at Dukes' Stages A and B, and those at Stage C. The former gave more positive reactions than the latter, but the difference was not significant.

The leucocytes from non-cancerous patients showed a strong reaction with foetal colon extract. Their mean MI was significantly lowered for every antigen concentration (Table). In fact, a strong inhibition was observed in many cases (7/16 for the dilution at 500 μg/ml) irrespective of diagnosis (sigmoiditis, Crohn's disease, ulcerative colitis, etc.) and for all or almost all the antigen concentrations. Stimulation was rare. In one case, stimulation was observed for low concentrations and inhibition for high concentrations of foetal colon extract. The reverse was seen in another case.

Our data show an important difference between cancerous and non-cancerous leucocytes. In opposition to our working hypothesis, the former were only weakly sensitized and in a limited number of cases (30% for the 500μg/ml concentration, which was the only one to give significant results). Stimulation of leucocyte migration was the more frequent reaction. By contrast, leucocytes of non-cancerous patients were often strongly inhibited by foetal colon extract at various concentrations (similar results had been obtained by Bendixen (1969)). One can wonder whether this discrepancy was due to a difference in the intensity of leucocyte sensitization, or reactions to different antigens.

For some authors, such as Kjaer (1975) and Zöller *et al.* (1977) stimulation of leucocyte migration corresponds to a weak reaction, and inhibition to a stronger one, generally obtained with higher concentrations of the antigen extract. For example, in Zöller's experiments (Zöller *et al.*, 1977, 1979), the same extract at concentrations of 1 and 5 mg/ml provoked respectively a stimulation and an inhibition of leucocyte migration. In our studies, however, a 4-fold increase in the protein concentration of the antigenic extract (from 500 μg to 2 mg/ml) transformed stimulation into inhibition in only one case. Hence, to explain the different reactivity to foetal colon extract of cancerous and non-cancerous leucocytes, the 2-antigen hypothesis is not unlikely: one antigen would be responsible for the stimulation of cancerous leucocyte migration, the other for the inhibition of non-cancerous leucocyte migration.

We already proposed this 2-antigen hypothesis in our previous work (Burtin *et al.*, 1978) after showing that leucocytes of non-cancerous intestinal diseases reacted as much as those of cancer patients to HT29 tumour extract in the LMI test. This hypothesis was purely speculative, however, as the data did not favour it. By contrast, it is more attractive in our present study. One could even imagine that antigen(s) of the HT29 extract, that sensitized non-cancerous leucocytes, were also in the foetal colon. Hence, a separation of HT29 extract by elimination of foetal colon antigens would render LMI reaction more specific for cancerous leucocytes.

The poor reaction of cancerous leucocytes with foetal colon extract disagrees with the data given by experimental tumours of the intestine. The animals, mainly rats, bearing these tumours were seen to have antibodies (Martin *et al.*, 1976; Steele & Sjögren, 1974) and sensitized lymphocytes (Steele & Sjögren, 1974) reacting to antigens of the foetal intestine. Furthermore, Bansal *et al.* (1978) was able to protect rats against the graft of chemically induced intestinal tumours by immunization with syngeneic foetal colon and liver extract. In other studies, 2 categories of foetal antigens have been demonstrated, some organ-specific, and especially specific to intestine, others being common to several organs (Steele *et al.*, 1975).

The situation in human tumours is not so clear. However, Zöller *et al.* (1979) found leucocyte reactivity in patients with carcinomas of various organs (stomach, colon and lung) to human foetal extracts by the LMI method. These results are quite contrary to ours. Actually many differences exist between Zöller's experiments and ours. The main one lies in the type of extracts used in the tests: Zöller's extracts were prepared with 3M KCl from whole foetuses, and probably contained more antigens than our extracts obtained with saline from foetal colon only. That would mean that reactions observed by Zöller's group could be explained by the sensitization of cancerous leucocytes to non-organ-specific foetal antigens.

We are grateful to the physicians and surgeons who allowed us to obtain blood from their patients, and to the pathologists who made their files available to us, namely Prof. Loygue, Dr André, Dr Moreaux and Prof. Orcel. Foetuses used in this study were given us by the gynaecological ward of Hôpital Boucicaut, Paris (Head: Prof. Taurelle). Normal blood samples were obtained from the Blood Centre of Hôpital Paul Brousse, Villejuif (Head: Dr Subtil) and Hôpital Cochin, Paris (Head: Dr Bismuth). The statistical advice of Mrs Maunoury was very useful.

This work was partially supported by a grant from Institut National de la Santé et de la Recherche Médicale (A.T.P. 47-77-79 n° affectation 650 7480).

### REFERENCES

BANSAL, B. R., MARK, R., RHOADS, J. E. & BANSAL, S. C. (1978) Effect of embryonic tissue immunization on chemically induced gastrointestinal tumors in rats. I. Can embryonic antigens act as rejection antigens? *J. Natl Cancer Inst.*, **61**, 189.

BEAULIEU, R. (1976) Immunocancerology in solid tumors. VII. *Symp. Cancérologie de l'Université de Laval, Miami.*

BENDIXEN, G. (1969) Cellular hypersensitivity to components of intestinal mucosa in ulcerous colitis and Crohn's disease. *Gut*, **10**, 631.

BURTIN, P., CHANY, E., BEAULIEU, R., MAUNOURY, M. T., CHAVANEL, G. & SABINE, M. C. (1977) Use of a permanent cell line extract to study the tumor associated immune reactions in colorectal cancer patients by leucocyte migration inhibition test. *Cancer*, **39**, 2379.

BURTIN, P., PINSET, C., CHANY, E., FONDANECHE, M. C. & CHAVANEL, G. (1978) Leucocyte-migration-inhibition test in patients with colorectal cancer: Clinicopathological correlations. *Br. J. Cancer*, **37**, 685.

KJAER, M. (1975) The dose-related effect of tumor extract on the *in vitro* migration of leucocytes from patients with renal carcinoma. *Eur. J. Cancer*, **11**, 281.

MARTIN, F., MARTIN, M., LAGNEAU, A., BORDES, M. & KNOBEL, S. (1976) Circulating antibodies in rats bearing grafted colon carcinoma. *Cancer Res.*, **36**, 3039.

STEELE, G. & SJÖGREN, H. O. (1974) Embryonic antigens associated with chemically induced colon carcinomas in rats. *Int. J. Cancer*, **14**, 435.

STEELE, G., SJÖGREN, H. O. & PRICE, M. R. (1975) Tumor-associated and embryonic antigens in soluble fractions of a chemically-induced rat colon carcinoma. *Int. J. Cancer*, **16**, 33.

ZÖLLER, M., MATZKU, S. & SCHULTZ, U. (1977) Leucocyte migration studies in gastric cancer detection: An approach toward improved specificity and sensitivity. *J. Natl Cancer Inst.*, **58**, 897.

ZÖLLER, M., MATZKU, S., SCHULTZ, U. & PRICE, M. R. (1979) Sensitization of leucocytes of cancer patients against foetal antigens: leucocyte migration studies. *J. Natl Cancer Inst.*, **63**, 285.